# Methylene blue photodynamic therapy induces selective and massive cell death in human breast cancer cells

**DOI:** 10.1186/s12885-017-3179-7

**Published:** 2017-03-15

**Authors:** Ancély F. dos Santos, Letícia F. Terra, Rosangela A. M. Wailemann, Talita C. Oliveira, Vinícius de Morais Gomes, Marcela Franco Mineiro, Flávia Carla Meotti, Alexandre Bruni-Cardoso, Maurício S. Baptista, Leticia Labriola

**Affiliations:** Biochemistry Department, Chemistry Institute, University of São Paulo, São Paulo, 05508-000 SP Brazil

**Keywords:** Breast cancer, Photodynamic therapy, Methylene blue, Selectivity

## Abstract

**Background:**

Breast cancer is the main cause of mortality among women. The disease presents high recurrence mainly due to incomplete efficacy of primary treatment in killing all cancer cells. Photodynamic therapy (PDT), an approach that causes tissue destruction by visible light in the presence of a photosensitizer (Ps) and oxygen, appears as a promising alternative therapy that could be used adjunct to chemotherapy and surgery for curing cancer. However, the efficacy of PDT to treat breast tumours as well as the molecular mechanisms that lead to cell death remain unclear.

**Methods:**

In this study, we assessed the cell-killing potential of PDT using methylene blue (MB-PDT) in three breast epithelial cell lines that represent non-malignant conditions and different molecular subtypes of breast tumours. Cells were incubated in the absence or presence of MB and irradiated or not at 640 nm with 4.5 J/cm^2^. We used a combination of imaging and biochemistry approaches to assess the involvement of classical autophagic and apoptotic pathways in mediating the cell-deletion induced by MB-PDT. The role of these pathways was investigated using specific inhibitors, activators and gene silencing.

**Results:**

We observed that MB-PDT differentially induces massive cell death of tumour cells. Non-malignant cells were significantly more resistant to the therapy compared to malignant cells. Morphological and biochemical analysis of dying cells pointed to alternative mechanisms rather than classical apoptosis. MB-PDT-induced autophagy modulated cell viability depending on the cell model used. However, impairment of one of these pathways did not prevent the fatal destination of MB-PDT treated cells. Additionally, when using a physiological 3D culture model that recapitulates relevant features of normal and tumorous breast tissue morphology, we found that MB-PDT differential action in killing tumour cells was even higher than what was detected in 2D cultures.

**Conclusions:**

Finally, our observations underscore the potential of MB-PDT as a highly efficient strategy which could use as a powerful adjunct therapy to surgery of breast tumours, and possibly other types of tumours, to safely increase the eradication rate of microscopic residual disease and thus minimizing the chance of both local and metastatic recurrence.

**Electronic supplementary material:**

The online version of this article (doi:10.1186/s12885-017-3179-7) contains supplementary material, which is available to authorized users.

## Background

Breast cancer is a worldwide health problem for women, it is the first in incidence and the second in mortality among all cancer types, even with all recent technological advancements [1]. Early intervention is impactful, but a large number of patients still relapse even after years of apparent cure. The challenges in combating the disease relies on the intrinsic tumour resistance properties, molecular heterogeneity, and metastasis [1–3]. The molecular subtypes of breast cancers are defined based on the presence of oestrogen receptors (ER), progesterone receptors (PR), and human epidermal growth factor receptor-2 (HER2). About 20% of breast cancers are negative for ER, PR and HER2 expression (Triple-Negative Breast Cancer; TNBC) displaying aggressive pathological features and high rates of metastasis and recurrence [4–6]. For TNBC patients, the only current option is a non-targeted chemo and/or radiotherapy in order to extend the survival of patients, but does not reliably prevent a secondary disease [7].

Photodynamic therapy (PDT) is a promising alternative treatment for controlling malignant diseases [8–10]. PDT is based on the photooxidation of biological matter; the treatment involves the uptake of a photosensitizer (Ps) followed by illumination with light of an appropriate wavelength that is able to excite the Ps and trigger photochemical reactions that generate reactive oxygen species, such as singlet oxygen (^1^O_2_), and radicals that lead to cell death [11]. The advantages of PDT compared with surgery, chemotherapy, or radiotherapy are the reduced long-term morbidity and the fact that PDT does not compromise other treatment options [11]. This therapy has been used as an experimental treatment modality in many countries for a number of cancers [12, 13]. In particular for non-superficial tumours, PDT appears promising in the treatment of high recurrence types of cancer. Indeed, it has been recently shown that PDT in combination with surgery in orthotopically implanted human pancreatic cancer in a nude mouse model was highly effective in eliminating microscopic disease in the post-surgical tumour bed as well as in preventing local and metastatic recurrence [14, 15].

For a variety of reasons, which include lack of studies on its efficacy and safety, as well as detailed mechanistic information, PDT is not a common type of treatment [12, 16, 17]. To overcome this scenario, many studies using PDT focus on the enhancement of Ps efficiency or in developing target-based PDT [18, 19]. However, because of the complexity of biological systems and unknown possible biological targets, details of how PDT operates are still elusive [13, 20]. Several approaches have also been developed using phenothiazinium derivatives, such as methylene blue (MB), as a new treatment strategy, leading to a PDT protocol which is efficient and also inexpensive [21–25]. In addition to the low cost and commercial availability, the use of MB is also interesting because it has been safely used for decades in other clinical applications [21, 22, 26, 27].

In this study, we set out to explore the effectiveness of PDT using MB as Ps (MB-PDT) in different human breast cell lines, as well as the molecular mechanisms related to cell death. We demonstrated that MB-PDT is selective in inducing massive cell destruction of malignant cells, especially TNBC cells. We also observed that apoptosis is not the predominant route of cell death induced by MB-PDT. Finally, by using a tridimensional (3D) cell culture model, we confirmed the effectiveness of MB-PDT in selectively eliminating tumour cells while not affecting normal-like cells.

## Methods

### Cell cultures

Non-tumorigenic human MCF-10A (ATCC CRL-10317™) breast cell line was maintained in phenol red-free Dulbecco’s Modified Eagle’s Medium/Nutrient F-12 Ham (DMEM-F12; Sigma-Aldrich, St. Louis, MO, USA) supplemented with 5% heat-inactivated horse serum (Vitrocell Embriolife, Campinas, Sao Paulo, Brazil), insulin (10 μg/ml; Sigma-Aldrich), cortisol (500 ng/ml; Sigma-Aldrich), cholera enterotoxin (100 ng/ml; Sigma-Aldrich), and epidermal growth factor (20 ng/ml; Sigma-Aldrich). Human breast adenocarcinoma cell line MCF-7 (ATCC HTB-22™) was maintained in phenol red-free Dulbecco’s Modified Eagle’s Medium/Nutrient F-12 Ham (DMEM-F12; Sigma-Aldrich) supplemented with 10% heat-inactivated foetal bovine serum (FBS) (Vitrocell Embriolife). Human breast adenocarcinoma cell line MDA-MB-231 (ATCC HTB-26™) was cultured in phenol red-free Roswell Park Memorial Institute Medium Modified (RPMI 1640; Sigma-Aldrich) supplemented with 10% FBS (Vitrocell Embriolife). All cultures were maintained at 37 °C under water-saturated atmosphere containing 5% CO_2_. For the 3D culture assays, (2x10^4^/cm^2^) cells were seeded on top of lamin-rich extracellular matrix gels (lrECM);commercially available as Matrigel (BD Biosciences, San Jose, CA, USA)- in phenol red-free medium supplemented with 2.5% serum and 5% lrECM and maintained for four days after treatments [28]. All 2D assays were also performed in 2.5% serum-supplemented medium.

### Photodynamic treatment

Phenotiazonium salt, MB (Labsynth Products, São Paulo, Brazil) was used as Ps to perform the PDT treatment. Cells were incubated for 2 h with 0.2, 2 or 20 μM MB, in phenol red-free medium supplemented with 2.5% FBS and maintained in these conditions during both irradiation as well as post-treatment times (1, 3 and 24 h). The whole microplate was irradiated with a light emitting diode (LED) array, with maximum emission wavelength at 640 nm, corresponding to total light doses of 4.5 J/cm^2^. Control experiments such as cells neither exposed to the Ps nor to light (control); cells not exposed to the Ps but washed and exposed to light (phototoxicity); and cells exposed to the Ps alone without irradiation (dark toxicity) were performed in all experiments.

### Cell viability assay and morphological studies

4 × 10^4^ cells/cm^2^ were plated and maintained in control conditions or exposed to MB-PDT and then stained with the DNA-binding dyes Propidium iodide (PI, Sigma-Aldrich) and Hoechst 33342 (HO, Sigma-Aldrich) for 10 min. Following incubation, the percentage of viable and dead cells was determined using an inverted fluorescence microscope (Nikon Eclipse Ti, Kyoto, Japan) with 20x of magnification. Tri-dimensional cell cultures were transferred to a glass slide and visualized using a confocal microscope (Axiovert 200 LSM 510 Laser and Confocor Modules, Carl Zeiss, Göttingen, Germany) equipped with water immersion objective (40X). Fluorescence of labelled cells was detected using laser 461 nm and 545 nm for excitation of HO and PI respectively. The cultures were evaluated according to: the total number of cells, determined by counting the nuclei stained with HO; and the number of dead cells determined by the number of nuclei stained with PI or by brightly HO (condensed chromatin) [29]. A minimum of 500 cells was counted in each experimental condition. Results were expressed as percentage of dead cells.

### Intracellular methylene blue quantification

1 × 10^5^ cells/cm^2^ were plated and incubated with 5 mL of medium containing MB (20 μM) and maintained for 1, 2, 4, 6 and 8 hours. At each time point, the supernatant was discarded and the cells were washed twice with PBS and then 1 mL of 50 mM SDS was added to promote lysis of the cell membrane. The supernatant was collected and absorbance was measured at the wavelength of maximum absorption of the MB solution used (655 nm). The incorporation of MB was determined by correcting the absorbance of MB by the number of cells remaining in each well after the incubation period.

### Intracellular singlet oxygen generation

Singlet oxygen measurements were performed in a specially designed Edinburgh F900 instrument (Edinburgh, UK) consisted of a Rainbow OPO (Quantel Laser-France) 10Hz, 2 mJ/pulse, which was pumped by a Brilliant Nd-YAG laser (Quantel Laser-France) and equipped with a cuvette holder, a silicon filter, monochoromator, a liquid-nitrogen-cooled NIR PMT (R5509) (Hamamatsu Co., Bridgewater, NJ, USA) and a fast multiscaler analyser card with 5 ns/channel (MSA-300; Becker & Hickl, Berlin, Germany). The cells were seeded in six-well plates (4x10^5^ cells/well) and after 24 h were incubated with MB for 2 h. The cells were washed in PBS, removed from the plates using trypsin solution, centrifuged and suspended in D_2_O saline solution and were directly excited at 664 nm inside a fluorescence quartz cuvette. We obtained ^1^O_2_ emission spectra by measuring emission intensities from 1200 to 1348 nm with 1 to 5 nm steps. The intensities of the near infrared (NIR) emission peak (centred at 1275 nm) are correlated with the amount of ^1^O_2_ generated.

### Glutathione quantification

Reduced glutathione (GSH) was quantified as previously described by Kand’ár et al [30] with minor modifications. Cells were seeded in Petri dishes (100 mm) at an initial density of 2.6 × 10^6^ cells/Petri dish. After 48 h the cells were washed in PBS, removed from the plates using 0.1% trypsin, centrifuged and suspended in deionized water. An aliquot was separated for cell count before lysis with 0.15% polidocanol (Sigma-Aldrich). For protein precipitation, samples were incubated with cold 10% metaphosphoric acid (10 min, 4 °C), centrifuged (22,000 x g, 15 min, 4 °C) and supernatants were collected. The supernatant was diluted 20 folds in 100 mM phosphate buffer pH 8.9 containing 0.1 mM diethylene triamine pentaacetic acid (DTPA, Sigma-Aldrich). Twenty microliters of this sample were diluted to 320 μL in a 100 mM phosphate buffer pH 8.0 containing 0.1 mM DTPA. Derivatization was performed by adding 20 μL of 148 mM orthophthaldehyde (OPA, Sigma-Aldrich) to this solution. The reaction was incubated at 25 °C for 15 min in dark. The samples were then filtered through a 0.22 μm polyvinyl difluoride (PVDF) filter and injected onto high performance liquid chromatography (HPLC). The GS-OPA product was separated in a VP-ODS/C8/Phenyl column (250 mm × 4.6 mm × 4.6 μm, Shimadzu, Kyoto, Japan). The HPLC was equipped with two LC-20AT solvent delivery systems, SIL-20 AC HT autosampler, CTO-20HC column oven, RF-20A fluorescent detector and CBM-20A system controller (Shimadzu, Kyoto, Japan). The separation was achieved using an isocratic elution of 15% methanol in 85% 25 mM Na_2_HPO_4_ (Merck, Darmstadt, German) pH 6. The flow rate was constant at 0.5 mL/min 37 °C. Fluorescent GS-OPA was monitored with a ʎ_ex_ 350 nm and ʎ_em_ 420 nm. The peak area was plotted against an external GS-OPA standard curve previously derivatized with pure GSH (Sigma-Aldrich). Results were presented as pmol/cell.

### Intracellular MB localization

We used confocal microscopy to characterize the subcellular localization of MB. To this end, we compared the fluorescence arising from cell cultures simultaneously incubated in the presence of MB and standard fluorescent markers of organelles. MitoTracker Green (Invitrogen, Paisley, UK) was used as a mitochondrial marker, LysoTracker Green (Invitrogen) as a lysosome marker and HO as a marker for the cell nucleus. Confocal images were taking using a laser scan microscope (LSM) - 510 from Zeiss using 1.2 N.A. 40x water immersion or 1.4 N.A. 63x oil immersion objective lenses. The laser and filter settings were: laser lines for MB = 633, Lyso = 488 and Hoechst 33342 = 364; beam splitter = HFT UV/488/543/633; emission filters for MB: 651-704, Lyso = 501-554 and Hoechst 33342 = 435-485. The imaging settings were: zoom = 1, dimensions = 512x512 pixels, image depth = 16 bit, averaging signal = 2 and optical section thickness = 2 μm. Images had their brightness and contrast adjusted for the figures and were analysed with ImageJ Software (National Institutes of Health).

### Detection of acidic vesicles in live cells using acridine orange

Acridine orange (AO) is a weak base that can accumulate in acidic spaces and emits bright red fluorescence. The intensity of the red fluorescence is proportional to the pH of the cellular acidic compartments [31]. In order to detect and quantify acidic vesicle formation during the process of autophagy, 4x10^4^ cells/cm^2^ were plated and then subjected or not to MB-PDT. Cells were then washed with PBS and stained with AO (Sigma Aldrich) at a final concentration of 5 μg/ml for 10 min at 37 °C in the dark. After a washing step, live cells were visualized using an inverted microscope for transmitted light and epifluorescence (Axiovert 200, Carl Zeiss) equipped with a C-APOCHROMAT 40×/1.20 M27 objective (Zeiss™). Fluorescence of AO-stained vesicles was detected by using a filter set 09 (Zeiss™) that provides an excitation band pass (BP) of 450-490 nm with emission long pass (LP) of 515 nm.

### Inhibition of signaling pathways

4.0 × 10^4^ cells/cm^2^ were plated and incubated with each inhibitor in the presence or in the absence of MB for 2 h, in a 5% CO_2_ humidified atmosphere at 37 °C. Cells were then subjected or not to light irradiation as described above. Apoptosis was inhibited by using a specific caspase-3 inhibitor (100 nM *Caspase-3 Inhibitor VI*; Calbiochem, La Jolla, CA, USA), a pan-caspase inhibitor (20 μM Z-VAD-FMK; Calbiochem) or a BAX inhibitor (10 μM; Tocris, Ellisville, MO, USA). Autophagy was activated using mTOR inhibitor rapamycin (20 nM; Cell Signaling Technology, Danvers, MA, USA) or inhibited by using either PI3-kinase inhibitor LY294002 (50 μM; Tocris), class III PI3K inhibitor 3-MA (5 mM; Calbiochem), chloroquine (5 μM; Sigma-Aldrich) or bafilomycin A1 (50 nM; Calbiochem). We analysed the dose-response and toxicity of each inhibitor and we used the highest concentration that presented no cytotoxicity in the control conditions for each cell line.

### Transient oligonucleotide transfection

The siRNA used in this study was a Silencer pre-designed siRNA (Invitrogen) of sequence 5’GCUCUGCCUUGGAACAUCAtt 3’. “AllStars negative control siRNA” (Qiagen, Venlo, Netherlands) was used as a negative siRNA control of scrambled sequence (siCTR). Transfection of siRNA was done using the lipid carrier Lipofectamine RNAiMAX (Invitrogen). Lipid-RNA complexes were formed in Opti-MEM (Invitrogen) in a proportion of 0.6 μl of Lipofectamine to 0.45 μl of 20 μM siRNA, at room temperature for 20 min and were further added to cells in antibiotic-free medium to reach a final volume of 300 μl for overnight transfection. Cells were maintained in culture for a 24-h recovery period before experiments were carried out. The efficiency of transfection/silencing was validated by Western blot, with at least 60% of inhibition.

### Western blots

Total protein extracts were prepared from each culture subjected to the treatments described above. Equal amounts (100 μg) of protein from each extract were solubilized in sample buffer (60 mM Tris-HCl [pH 6.8], 2% SDS, 10% glycerol, 0.01% bromophenol blue) and subjected to SDS-PAGE (16%). Proteins were transferred to PVDF membranes, incubated with Blocking Buffer solution (Thermo Fisher Scientific) and 5%BSA 1:1, and then incubated with the following antibodies: rabbit polyclonal anti-BAX (2772), rabbit polyclonal anti-BCL2 (2876) (all from Cell Signaling Technology), rabbit polyclonal anti-LC3 (L8918) and mouse monoclonal anti-alpha-tubulin clone B-5-1-2 antibody (T5168) (all from Sigma-Aldrich) as a loading control. Membranes were then incubated with horseradish peroxidase-conjugated secondary antibody (Vector Laboratories, Burlingame, CA, USA). Enhanced chemiluminescence was performed according to the manufacturer’s instructions (Amersham Biosciences, Little Chalfont, UK). Quantitative densitometry was carried out using the ImageJ software (National Institute of Health [NIH]). The volume density of the chemiluminescent bands was calculated as integrated optical density × mm^2^ after background correction.

### Caspase-3, caspase-7, caspase-8 and caspase-9 activity assays

4 × 10^6^ cells were collected and caspase activity was measured using a specific fluorimetric assay (BioVision Research Products, Mountain View, CA, USA). The reactions were started at 37 °C by incubating 50 μg of total protein extracts with a specific caspase substrate (50 μM DEVD-AFC, 50 μM IETD-AFC or 50 μM LEHD-AFC for caspase-3 and -7; -8 and -9, respectively; BioVision), following the manufacturer’s instructions. Protease activity was evaluated at an excitation wavelength of 400 nm and an emission wavelength of 505 nm using a 96-well plate spectrofluorometer (Spectra MAX M2; Molecular Devices, Sunnyvale, CA, USA). Total protein extracts from Min6 cells exposed to either vehicle or a combination of pro-inflammatory cytokines which induced a significant and well documented degree of apoptosis [32–35], were always included as a positive control for caspase-3, -7, -8 and -9 activity assays, in each experiment performed. Caspase-3, -7, -8 and -9 activities were expressed as arbitrary fluorescence units per 50 μg of total protein (At least 3 independent experiments were performed in triplicate for each condition).

### Statistical analysis

All results were analysed for Gaussian distribution and passed the normality test. The statistical differences between group means were tested by One-way ANOVA followed by Tukey post-test for multiple comparisons. A value of *p* < 0.05 was considered as statistically significant.

## Results

### MB-PDT selectively induces cell death in breast cancer cells, whereas not significantly affecting non-malignant cells

**Fig. 1:**
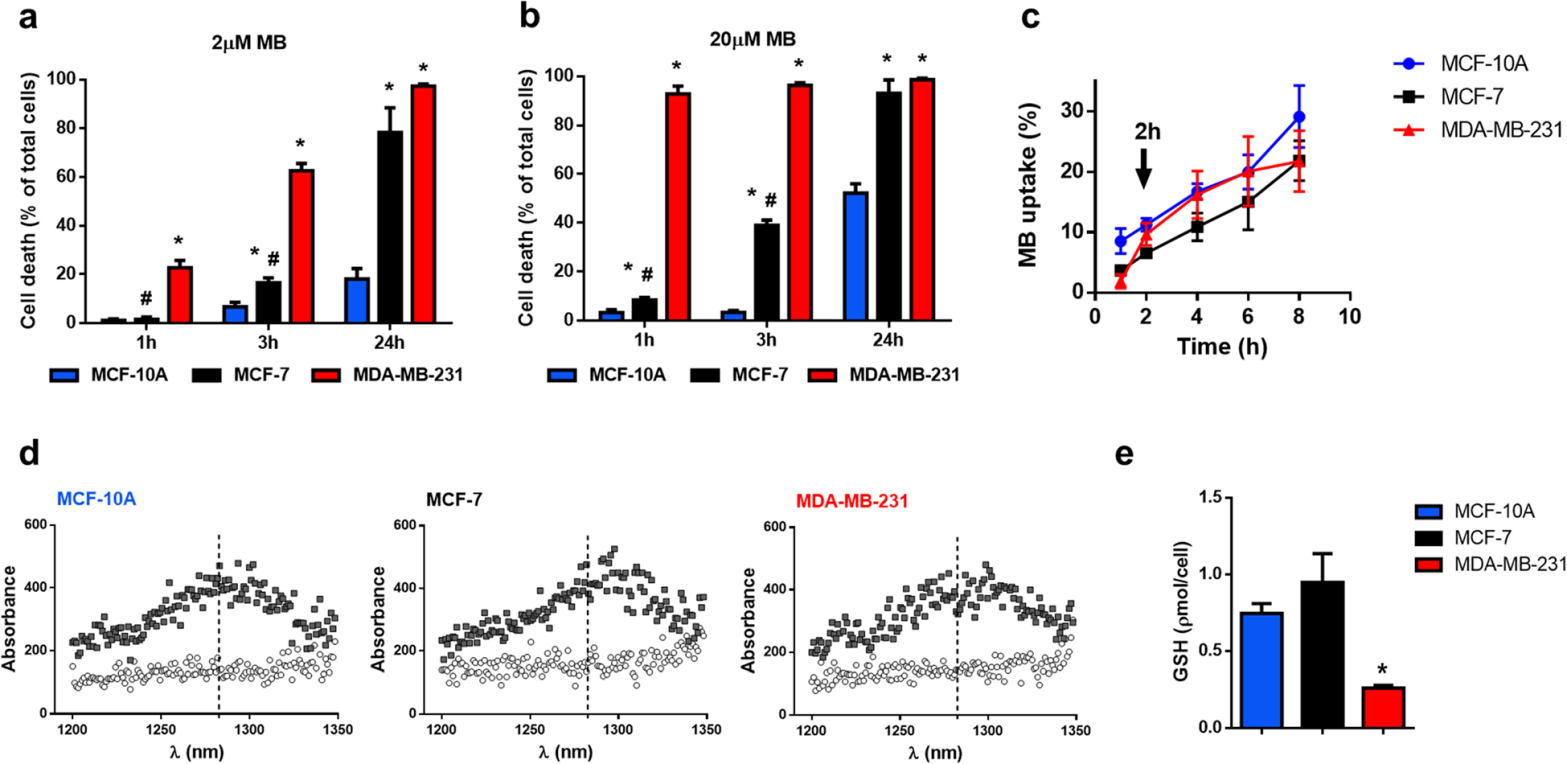
MB-PDT induces massive death in tumorigenic cells and weakly affects normal-like cells. Viability time curves after MB-PDT of cell cultures with 2 (**a**) or 20 μM of MB (**b**) followed by 4.5 J/cm^2^ irradiation obtained at 1 h, 3 h and 24 h post-irradiation (*n* = 3 independent experiments) * *p* < 0.05 *versus* MCF-10A; # *p* < 0.05 *versus* MDA-MB-231. (**c**) Curves of MB incorporation in MDA-MB-231, MCF-7 and MCF-10A after 1, 2, 4, 6, and 8 h of incubation (*n* = 4 independent experiments). (**d**) Emission spectrum from MB-free MCF-10A, MCF-7 and MDA-MB-231 (white circles); and emission spectra from cells exposed to 20 μM MB for 2 h (*gray squares*). (**e**) Cellular GSH levels in MDA-MB-231, MCF-7 and MCF-10A cells * *p* < 0.05 *versus* MCF-10A. Results are shown as mean ± s.e.m

Taking into account the heterogeneity of the most common breast cancer types and also to test the possible cytotoxic effects of MB-PDT in normal-like cells, we used the following human breast epithelial cell lines: MCF-7, an ER, PR and HER-2-positive, luminal A cell line; MDA-MB-231, a TNBC cell line; and MCF-10A, a normal-like cell line. Figure 1 (a and b) shows time curves of cell death after MB-PDT with 2 or 20 μM MB followed by irradiation with 4.5 J/cm^2^. The treatment consistently had a higher impact in the malignant cell lines and presented a maximal effect 24 h after irradiation in the presence of 20 μM MB. The TNBC cells showed the highest rate of cell death (24 h: 98.6% ± 0.5%), followed by MCF-7 cells (93.0 ± 5.2%) and then by the normal-like MCF-10A cells (52.2% ± 3.8%). Additionally, unlike the exponential increase in cell death over time presented by the other cell lines, in the presence of the highest MB concentration, MDA-MB-231 cells reached the maximal percentage of photodynamic destruction at earlier time points (1 h).

Using the lower concentration of MB, we detected that the normal-like cells were even less sensitive to MB-PDT (24 h: 18.0% ± 7.2%). It is important to note that this dose still induced massive death in the malignant cell lines at the same time point (MDA-MB-231: 97.3% ± 0.7% and MCF-7: 78.3% ± 7.1%). These data allowed us to establish a window of time for our mechanistic studies. It is important to note that cells submitted to irradiation alone (without MB) or MB alone up to 24 h of incubation (to test dark toxicity) showed no significant differences in cell death in comparison to untreated cells. Moreover, survival of all cell lines exposed to different MB concentrations or light alone was similar to the values obtained for the negative control conditions (see Additional file 1: Figure S1).

To analyse whether the distinct susceptibility to MB-PDT was due to differences in MB uptake, we measured the intracellular levels of MB and observed no statistical differences in the Ps content among all cell lines (Fig. 1c). We also assessed ^1^O_2_ generation capability and detected similar levels of this oxidant molecule between all cell lines (Fig. 1d). These results led us to conclude that the lower effect of MB-PDT was neither due to intracellular concentrations of the Ps nor to the amount of intracellular singlet oxygen. To evaluate if there was any differential stress-adaptive response to MB-PDT, we measured intracellular glutathione and found lower reduced glutathione (GSH) levels in MDA-MB-231 cells (Fig. 1e). This indicates that glutathione-dependent stress-control mechanism might be important to determine the sensitivity to the prooxidant milieu generated by MB-PDT.

### Relevance of apoptosis in MB-PDT-induced cell death

**Fig. 2:**
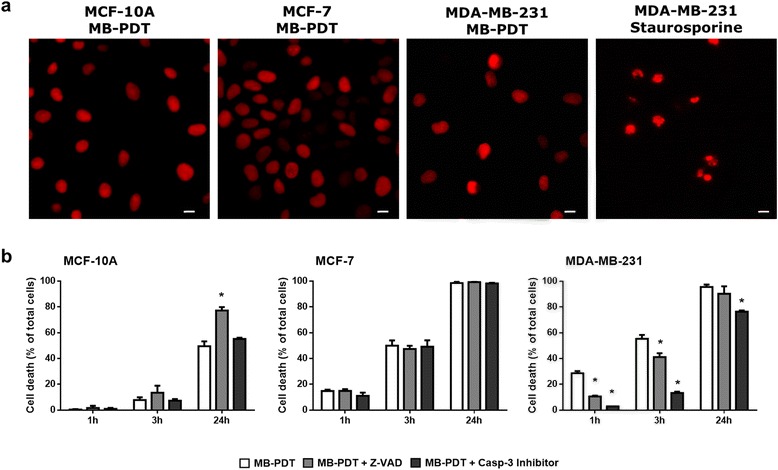
Apoptosis pathway is not the main mechanism involved in MB-PDT cell death. (**a**) Representative image of human mammary cells nuclei treated with MB-PDT or staurosporine (MDA-MB231 cells) stained with propidium iodide. Scale bar: 20 μm (**b**) Cell viability time curves obtained upon 1 h, 3 h and 24 h post MB-PDT performed in the presence or in the absence of a pan-caspase inhibitor (zVAD) or a caspase-3 specific inhibitor (*n* = 3 independent experiments) * *p* < 0.05 *versus* MB-PDT. Results are shown as mean ± s.e.m

We analysed the typical morphological changes related to cell death in the nuclei after treatment. MB-PDT did not induce neither the pyknotic and fragmented nuclei or condensation of chromatin into small, irregular and circumscribed patches, typical patterns of apoptotic cells in any time point or MB concentration tested (Fig. 2a, and see Additional file 1: Figure S2). As a control for typical apoptotic nuclei morphology, MDA-MB-231 cells were treated with the known apoptotic inducer staurosporine [36, 37]. The differences between typical morphology of nuclei undergoing apoptosis displayed by staurosporine-treated cells and the one displayed in MD-PDT-treated cells, led us to hypothesize that MB-PDT induced death through a non-apoptotic route.

**Fig. 3:**
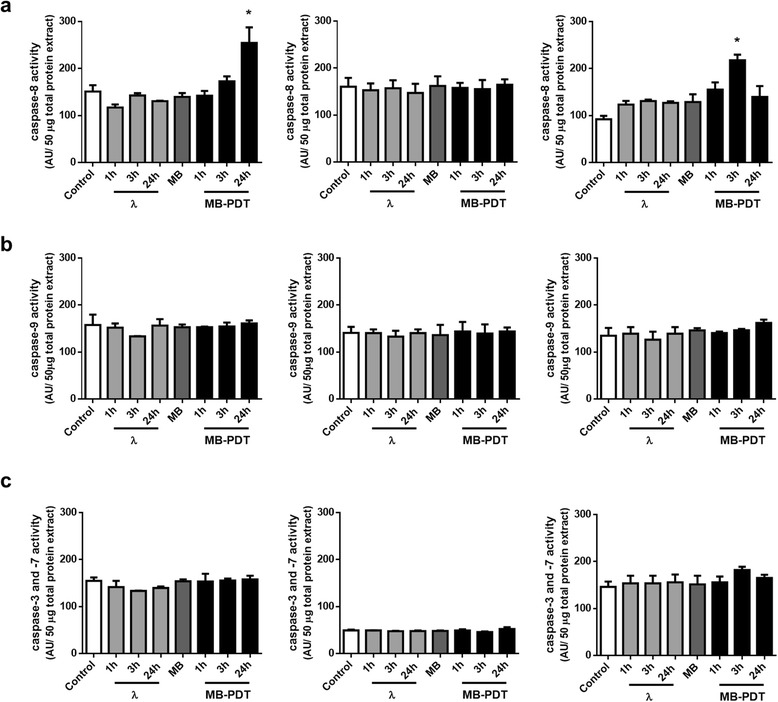
MB-PDT-induced cell death is independent of caspase activity. MDA-MB-231, MCF-7 and MCF-10A cells were untreated (control), only irradiated (λ), only incubated with MB (MB) or treated (MB-PDT). After 1 h, 3 h or 24 h of irradiation, cells were collected and lysed in an appropriate buffer. (**a**) Caspase-8, (**b**) caspase-3 and -7 and (**c**) caspase-9 activities were measured by a fluorimetric assay using a specific substrate utilizing 50 μg of total protein lysates. (*n* = 3 independent experiments). * *p* < 0.05 *versus* respective negative control. Results are shown as mean ± s.e.m

However, according to the current classification of cell death subroutes, only the presence of specific morphological features is not sufficient to establish which mechanism is mediating cell deletion [38, 39]. Thus, we also evaluated biochemical hallmarks of apoptosis. We analysed viability curves upon MB-PDT in the presence of either Z-VAD-FMK, a pan-caspase inhibitor, or of a specific caspase-3 inhibitor (Fig. 2b). Strikingly, our results demonstrated that both inhibitors exerted a cytoprotective effect at initial times after MB-PDT, but failed to completely prevent cell death in malignant cells. We also assessed the activity of initiator caspases-8 and -9, as well as executioner caspase-3 and -7 (Fig. 3). Corroborating the inhibition assays, no caspase-3 involvement was detected in MB-PDT-induced cell death in either MCF-10A or MCF-7 cells. However, a significant transient peak of caspase-8 activity was observed at initial times after MB-PDT in MDA-MB-231 cells and in MCF-10A cells at 24 h after treatment.

**Fig. 4:**
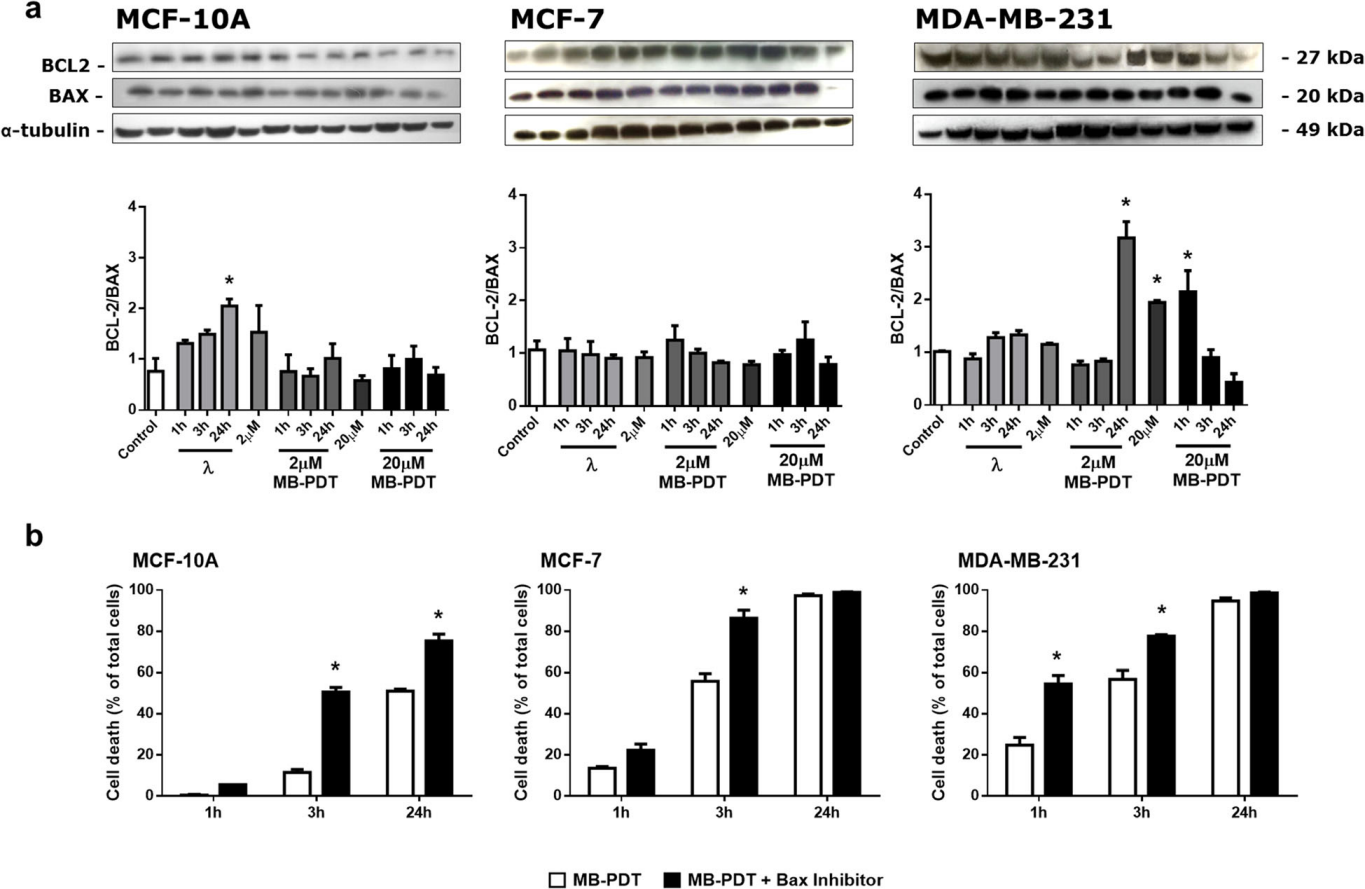
Caspase-independent pathway of apoptosis does not interfere with cell fate upon MB-PDT. (**a**) WB analysis of BCL2/BAX ratio in MDA-MB-231, MCF-7 and MCF-10A cells subjected or not to MB-PDT upon 1 h, 3 h or 24 h post-irradiation. Immunoblots shown are representative results. The corresponding bar graph results from densitometry analysis from all blots. Results are presented as mean ± s.e.m., (*n* = 3 independent experiments); * *p* < 0.05 *versus* control. (**b**) Viability time curves obtained after 1 h, 3 h and 24 h post MB-PDT in the presence or absence of a specific pharmacological BAX inhibitor (*n* = 3 independent experiments) * *p* < 0.05 *versus* MB-PDT. Results are shown as mean ± s.e.m

These results led us to propose that a caspase-independent apoptotic pathway could mediate MB-PDT-induced cell deletion. Therefore, to further determine the relevance of the apoptosis pathway after MB-PDT, we evaluated the balance between anti- and pro-apoptotic proteins of the B-Cell CLL/lymphoma 2 (BCL2) family. As shown in Fig. 4a, none of the experimental conditions tested induced a decrease in BCL2 and BCL2-associated X protein (BAX) protein ratio. We also tested the effect of a BAX specific inhibitor on MB-PDT efficiency (Fig. 4b). Cell viability revealed that the inhibition of BAX pore formation is harmful for all cell lines, which can then become more susceptible to MB-PDT. Altogether these data presented evidence that the caspase-independent apoptotic pathway had no relevance in MB-PDT-induced cell damage.

### MB fluorescence concentrates at the lysosomes of breast cancer cells

**Fig. 5:**
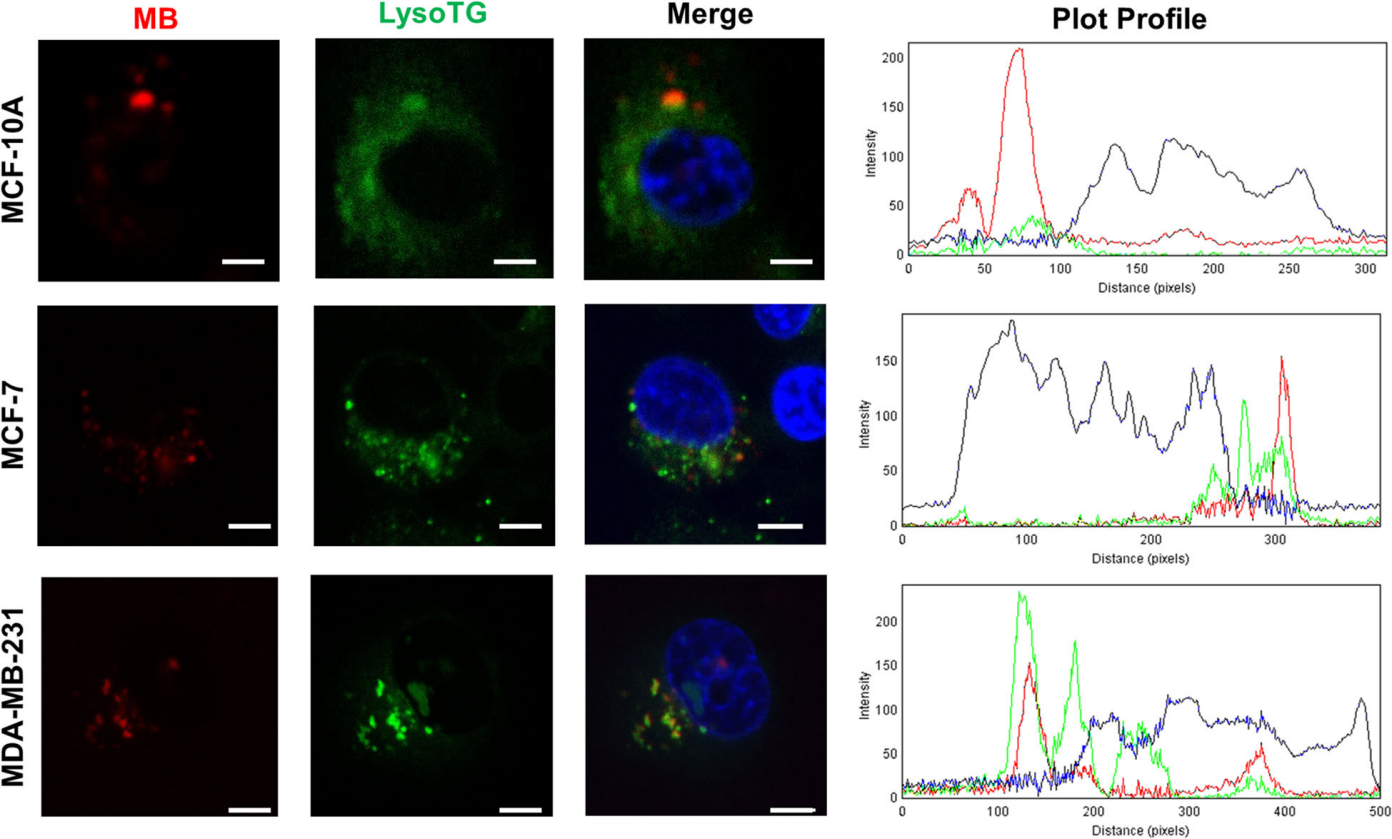
MB localizes in the lysosomes of MCF-10A, MCF-7 and MDA-MB-231 cells. Confocal microscopy images of cells simultaneously incubated with LysoTracker *green* (LysoTG, *green*), MB (*red*) and Hoechst 33342, for nuclei staining (*blue*). Plot profiles quantify the intensity of *red*, *green* and *blue* fluorescence from a straight line in the middle of the cell. [MB] = 20 μM; [LysoTG] = 300 nM; nucleus (HO 3334 = 300 nM). Scale Bar: 10 μm

To determine the subcellular localization of MB in organelles involved in cell death mechanisms, we incubated the cells with MB in combination with a nuclear marker and either a lysosomal (LysoTG) or a mitochondrial marker (MitoTG). All cell lines showed some level of colocalization of LysoTG and MB fluorescence signals. However, in the MDA-MB-231 cells, MB was highly concentrated at the lysosomes showing a near perfect overlap with LysoTG staining (Fig. 5 and see Additional file 1: Figure S3a). In sharp contrast, this pattern of colocalization was not observed for MB and MitoTG (see Additional file 1: Figure S3b). This finding represents a preferential lysosomal localization of MB, which makes this subcellular compartment prone to photochemistry damage induced by MB-PDT instead of the mitochondrion or the nucleus.

### MB-PDT-induced autophagy leads to an increase in cytoprotection only in MDA-MB-231 and MCF-10A

**Fig. 6:**
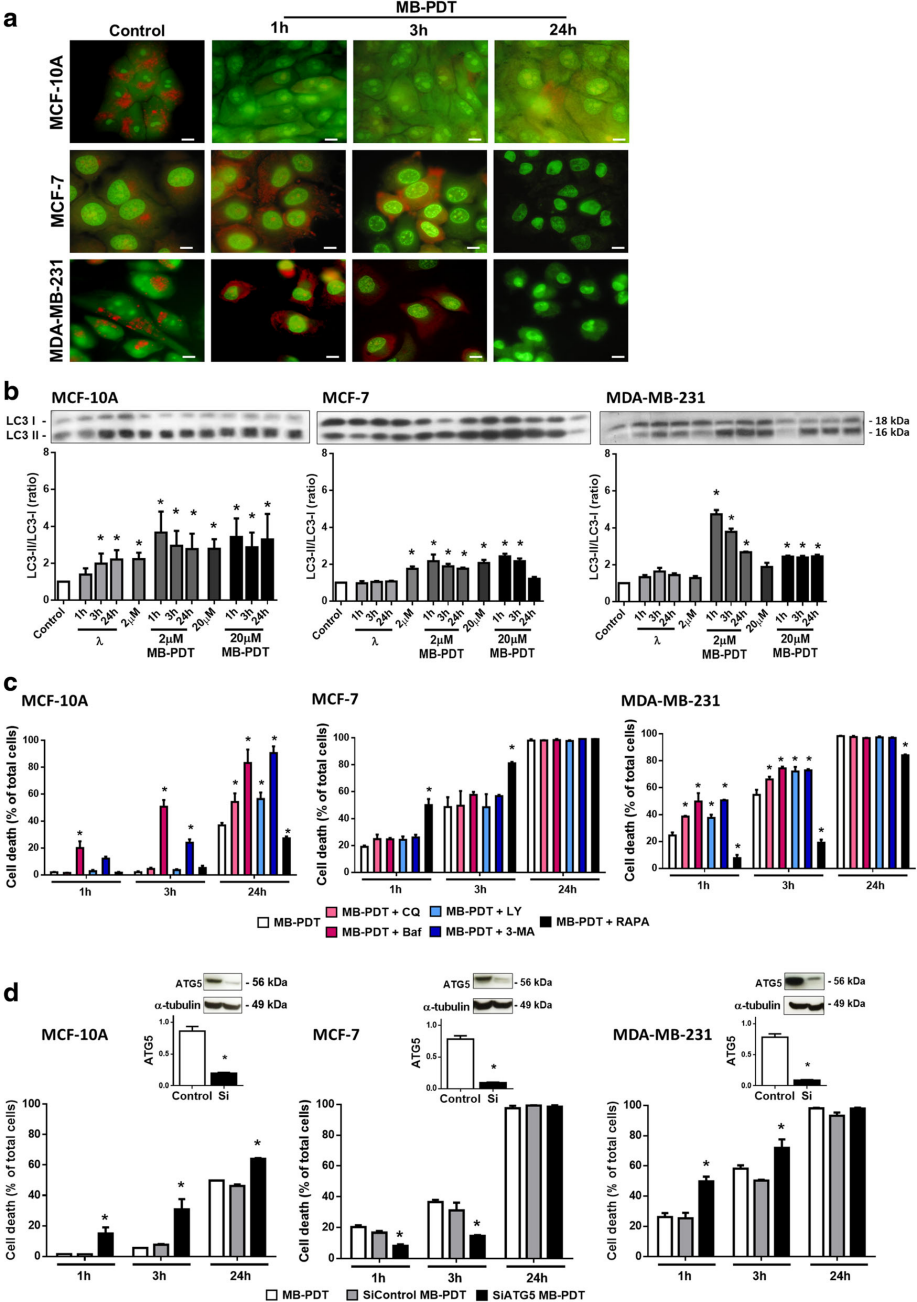
MB-PDT-induced autophagy leads to cytoprotection (MDA-MB-231 and MCF-10A) or cell death (MCF-7). (**a**) Representative images of acidic vesicle formation in cells subjected to MB-PDT after 1 h, 3 h or 24 h or not treated (control). Upper panels: MCF-10A; middle panels: MCF-7; and lower panels: MDA-MB-231. Scale bar: 10 μm. (**b**) WB analysis of LC3-II/LC3-I ratio after MB-PDT treated cells with 2 or 20 μM MB, irradiated only after 1 h, 3 h or 24 h, incubated with 2 or 20 μ MB in the dark or not treated (control). Immunoblots shown are representative results. The corresponding bar graph results from densitometry analysis from all blots. Results are presented as mean ± s.e.m., (*n* = 3); * *p* < 0.05 *versus* control. (**c**) Viability time curves with 2 μM (MDA-MB-231) or 20 μM (MCF-7 and MCF-10A) of MB obtained upon 1 h, 3 h and 24 h post-irradiation in the presence or in the absence of chloroquine (CQ), bafilomycin (Baf), LY294002 (LY), 3-MA or rapamycin (RAPA). (*n* = 3 independent experiments). * *p* < 0.05 *versus* MB-PDT. (**d**) Viability time curves of cells subjected or not to ATG5 silencing and then MB-PDT were obtained upon 1 h, 3 h and 24 h (*n* = 3 independent experiments). * *p* < 0.05 *versus* MB-PDT. Upper panels: WB analysis of ATG5 protein levels. Immunoblots shown are representative results. The corresponding bar graph results from densitometry analysis from all blots. Results are presented as mean ± s.e.m, (*n* = 3 independent experiments); * *p* < 0.05 *versus* control

A large number of different cell types initiate autophagy following photoirradiation [40]. Our results showed an increase in acidic structures already at early time points after MB-PDT in MDA-MB-231 cells (Fig. 6a). Moreover, a significant increase in LC3-II/LC3-I ratio was observed not only upon MB-PDT, but also after irradiation or MB incubation controls alone (Fig. 6b). In MDA-MB-231 cells, autophagosome formation was higher at initial times after PDT with 2 μM MB. These data showed autophagy was induced by the treatment, but it did not appear to be related to cell death. To determine whether the role of autophagy in MB-PDT is a mechanism of death or an attempt to rescue damaged cells, we assessed viability after MB-PDT by inhibiting or inducing autophagy. MDA-MB-231 and MCF-10A cells displayed a significant cell death increase upon autophagy inhibition with all inhibitors tested (Fig. 6c). In contrast, the autophagic flux induced by rapamycin decreased cell death in MDA-MB-231 and MCF-10A cells exposed to MB-PDT. This effect was not observed in MCF-7 cells, where an autophagy induction even resulted in increased cell susceptibility to MB-PDT. Consistent with these results, autophagy silencing by siRNA-mediated knockdown of ATG5 confirmed that this pathway elicits a cytoprotective role in MDA-MB-231 and MCF-10A but not in MCF-7 cells (Fig. 6d). In conclusion, our results indicated that autophagy might be related to an initial pro-survival response of the cells to the oxidative damage generated by MB-PDT in TNBC and normal-like cells, but not in luminal A cells.

### Spheroid culture enhances the differential sensitivity to MB-PDT of malignant and normal-like cells

**Fig. 7:**
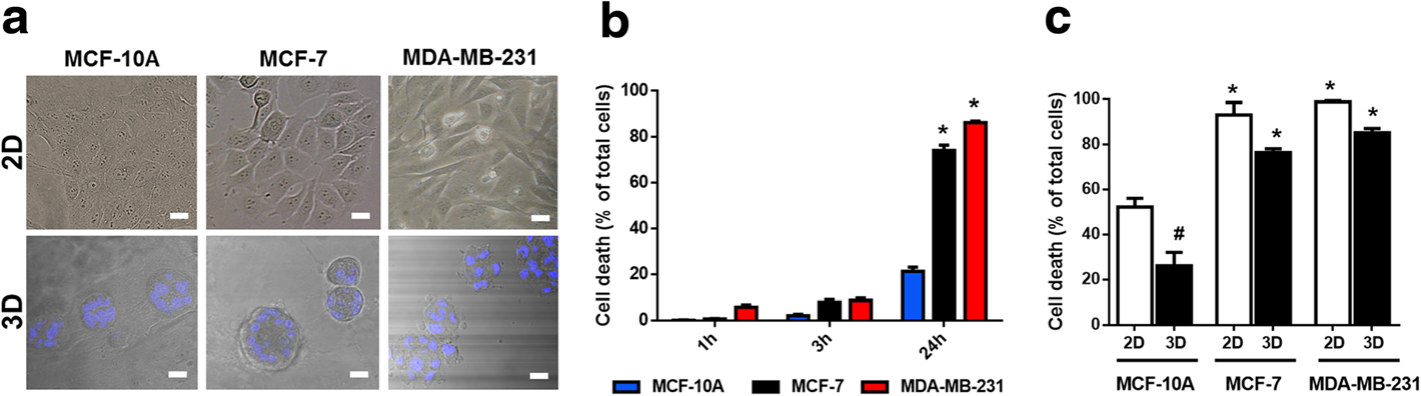
A tridimensional environment enhances the differential sensitivity between tumorigenic and normal-like cells. (**a**) Morphology of epithelial breast cells in different culture conditions. Upper panels: phase contrast micrographs of MDA-MB-231, MCF-7 and MCF-10A cells cultured in monolayer (2D); lower panels: confocal microscopy of MDA-MB-231, MCF-7 and MCF-10A cells cultured as spheroids (3D); nuclei were stained with Hoechst 33342 (blue); Scale bar: 20 μm. (**b**) Viability time curves after MB-PDT of 3D cultures with 20 μM of MB followed by of 4.5 J/cm^2^ irradiation obtained at 1 h, 3 h and 24 h post-irradiation (*n* = 3 independent experiments).* *p* < 0.05 *versus* MCF-10A. (**c**) Comparison of the MB-PDT cytotoxic effect between 2D and 3D cell cultures at 24 h post-irradiation. *p < 0.05 *versus* MCF-10A 3D; #*p* < 0.05 MCF-10A 2D *versus* MCF-10A 3D. (*n* = 3 independent experiments). Results are shown as mean ± s.e.m

In order to validate our results in a model that recapitulates the morphology of glandular epithelium, we compared the different responses in cells cultured on plastic surfaces (2D monolayers) or on top of a commercial basement membrane (3D). When cultured in a 3D environment, breast epithelial cells form multicellular structures; the normal-like cells organize into polarized, growth-arrested acini containing a lumen, whereas malignant cells form overgrown and disorganized tumour-like masses [28] (Fig. 7a). Thus, this strategy provides a more physiologically relevant assay to analyse the effect of treatments against malignant cells.

The same MB-PDT photocytotoxicity protocol used for cells grown on monolayers was performed in 3D cultures. Dose response curves using 20 μM MB followed by irradiation with 4.5 J/cm^2^ showed that there was no obvious cell viability inhibition both at 1 and 3 h after MB-PDT treatment. However, cell death was significantly increased after 24 h of PDT for both breast cancer cell lines (MDA-MB-231: 87.1% ± 1.8%; MCF-7: 74.4% ± 1.5%) (Fig. 7b). Importantly, MB-PDT effect in cancer cells cultured in 3D after 24 h did not differ significantly from the effect observed in 2D after MB-PDT, but normal-like cells cultured in 3D displayed a significantly lower sensitivity to the treatment than those cultured in 2D (Fig. 7c).

## Discussion

In this study we have demonstrated that MB-PDT induced massive cell death in two human breast cancer cell lines displaying different invasive properties. The highest sensitivity to MB-PDT was observed in MDA-MB-231 cells, used as a model of TNBC, a tumour subtype for which there are no targeted treatments. This effect is very relevant because TNBC tumours are the greatest challenge in breast cancer treatment nowadays [7]. With MB-PDT, we obtained almost 100% of cell death in these malignant cells. Our results are significantly better than the ones obtained in other reports using PDT for treating breast cancer cells [10, 41–43]. Other Ps used in previous studies, such as Mitoxantrone, present dark toxicity, which is not desired for PDT practice [12, 16]. In our study using MB we observed no dark toxicity, a feature which can increase local specificity and safety of the treatment.

One of the advantages of PDT over other cancer treatments is the possibility of generating less side effects to patients [8–10]. Our results demonstrated the efficacy of MB-PDT in selectively eliminating breast cancer cells. Among the few reports found in literature, Shemesh and collaborators have recently showed a small difference of only 20% in cell death between MFC-10A and MDA-MB-231 cells submitted to liposomal Indocyanine green-PDT [44]. In the present work, we have reached a difference of up to 80% of MB-PDT-induced cell death between malignant and normal-like cells.

Since it has already been demonstrated that MB is a classical ^1^O_2_ generator [45], we compared parameters like oxidative potential and antioxidative capability between the cell lines. The photodynamic action of MB leads to the production of similar levels of ^1^O_2_ in the intracellular microenvironment, so that is not a limitation for MB-PDT efficiency. As a prooxidant therapy, PDT is able to cause the collapse of antioxidant systems, leading to cell death [19]. Among the systems involved in the homeostasis of the intracellular redox balance, glutathione plays a major role [46]. It has been reported that TNBC cells present lower GSH levels compared to ER positive cells [47]; furthermore, cell lines containing low GSH levels tend to be much more sensitive to cancer therapies [46]. In this study we confirmed that TNBC cells present the lowest GSH intracellular levels among the studied cell lines, and that this could be related to the inability of these cells to cope with MB-PDT-induced oxidative stress. This result pointed MB-PDT as a potential strategy to effectively kill malignant cells that lack specific therapeutic targets by impacting metabolic properties that differ from those found in normal tissues.

There are few *in vitro* systematic studies on the molecular mechanisms induced by PDT that compare both malignant and normal-like mammary cells. These reports demonstrated that apoptosis is the main cell death pathway activated by PDT [48–51]. Nevertheless, we argue that in these studies classical characteristics of apoptosis have not been observed after PDT. Therefore, we hypothesize that apoptosis may not be the predominant process that mediates cell death induced by PDT, but only a by-product of other activated mechanisms [22]. Other authors also stated that the predominant type of cell death depends on the protocol adopted, and that there may be variations from apoptosis to necrosis depending, for example, on the energy dose used [11]. In our study we observed no presence of apoptotic traits under any dose, treatment time, or irradiation intensity used. Additionally, cell viability was not completely restored with the use of caspase inhibitors. Furthermore, MB-PDT action could not be exerted exclusively by apoptosis because MCF-7 cells do not express caspase-3 [52]. Additionally, no increase in caspase-7, -8 or -9 activities was detected in MCF-7 cells upon MB-PDT. In this context, we also observed no decrease in the ratio of anti- and pro-apoptotic proteins and that inhibition of the mitochondrial pore formation increased the sensitivity of the cells to MB-PDT, pointing to a probable mitochondrial-independent cell death pathway. Reinforcing this hypothesis, we showed that MB is not localized or, as already reported, it may be in a reduced state in mitochondria [45], thus strongly suggesting that this organelle is indeed not the primary target for MB-PDT oxidative damage.

Depending on a network of signals generated at specific cellular sites, cells can respond differentially to stress [53]. Previous studies exploring the subcellular localization of MB indicated that this Ps presents a tendency to accumulate in lysosomes of living cells [45]. We reported here for the first time evidence of differential patterns of lysosomes and MB colocalization in different epithelial cell lines of the same tissue. In view of the organelle-specific initiation of cell death, this data may contribute to explain the differential cellular sensitivity to MB-PDT observed among the three analysed cell lines.

The data on the lysosomal localization of MB, led us to explore the involvement of autophagy in the context of MB-PDT. The relationship between autophagy and cell death in PDT is still extensively discussed in the literature. Some studies pointed this pathway as responsible for the cell damage generated by different Ps [54, 55]. Others have shown that this type of cell death only occurs upon prevention of classical apoptosis, such as a result of mutations in essential apoptotic-related genes such as *BCL2* or caspase family members [40, 56]. Our data showed an intensification of the autophagic pathway upon MB-PDT which lead either to cytoprotection or cytotoxicity in a cell-dependent manner. This is expected since MCF-7 cells are haploinsufficient for Beclin-1 [57], which could be the reason for their increased sensitivity for MB-PDT.

The fact that cancer cells can die through different mechanisms is a relevant clue in the choice and design of anticancer therapies based on molecular targets [55]. We have found that, depending on the cell type, multiple cell death pathways might be activated upon MB-PDT. Indeed, we showed that apoptosis and autophagy are related, but not the main cell death inducing pathways. Moreover, preliminary results from our group are pointing some of the regulated necrosis pathways (i.e. necroptosis) as more relevant mechanisms involved in MB-PDT-induced cell death (data not shown). Since one important aspect for an alternative therapy for cancer treatment is to broaden the spectrum of cell death mechanisms to by-pass the different resistance mechanisms displayed by malignant cells, we are at the present time focusing our resources to further dissect the alternative molecular pathways leading to MB-PDT photocytotoxicity.

From an oncological point of view it is of fundamental importance if a therapy can achieve the ultimate goal of eliminating tumour cells, despite the complexity of the tumour microenvironment [58]. 3D cultures allows for the reestablishment of biochemical and morphological characteristics that resemble the conditions of the cells in their *in vivo* environment [28]. Since 3D cultures present the advantage of the formation of microenvironments and differential exposure to distinct factors such as nutrients and oxygen, this approach represents a robust model for the study of incorporation and bioactivity of drugs [59]. Moreover, it has been shown that 3D assays can be used successfully to effectively distinguish malignant and normal tissue responses to therapy [28, 60]. We have demonstrated that MB is efficiently incorporated in the complex gelatinous matrix used for the 3D cell cultures; it is able to enter the 3D structures and to be photoactivated inducing cell death isotropically throughout the spheroids. Our results indicate that in this model MB-PDT was also effective in inducing cell destruction of cancer cells, with an even higher selectivity between tumours and normal-like cells. This enhancement in MB-PDT preferential response in tumour cells might be due to the protective effect exerted by the organized basement membrane and tissue polarity seen in the acini formed by MCF-10A cells [61, 62].

In sum, MB-PDT holds promise as a useful adjunct to surgery to eliminate microscopic residual malignant cells in the post-surgical tumour bed and prevent local as well as metastatic recurrence without affecting normal tissues. In particular for breast cancer, PDT is not currently being tested as a peri-opertive therapy like the one described for pancreatic cancer (14-15); however, all results presented in this work pointed us the importance to pursue further studies in this direction in a near future. The strategy could be of particular importance in TNBC because metastatic recurrence after surgical tumour resection is a major and frequent cause of patient mortality.

## Conclusions

When compared to other studies of PDT on breast cancer, we have reached a greater efficiency in selectively killing cancer cells with no MB dark cytotoxicity. Besides, we demonstrated the differential effect of MB-PDT in more than one cell line, overcoming an important caveat in many PDT studies that use either only one tumour cell line or cell lines from different tissues. To the best of our knowledge, we have also shown for the first time that normal-like cells of breast epithelium are much more resistant to MB-PDT. These are fundamental features for the safe usage of the therapy, reinforcing the fact that one of the advantages of PDT over other therapeutic approaches is the possibility of generating fewer side effects to the patients. Finally, we propose that MB-PDT could be an effective and safe adjunct to surgery leading to lower rates of local and distant recurrence.

## Additional file

Additional file 1:
**Figure S1.** MB-PDT induced massive cell death in MDA-MB-231 without dark cytotoxicity. (**a**) Representative graph showing the viability of MCF-10A, MCF-7 and MDA-MB-231 cells after MB-PDT with 20 μM of MB followed by 4.5 J/cm^2^ irradiation and the experimental controls: irradiation alone (λ); dark toxicity of MB, that means incubation without irradiation (MB); or not treated cells (control). These results were obtained after 24 h (n = 3 independent experiments); *: p < 0.05 *versus* control. (**b**) Viability of MDA-MB-231 cells after MB-PDT with MB (20 μM) followed by 4.5 J/cm^2^ or 1.5 J/cm^2^ irradiation after 24 h. Results are shown as mean ± s.e.m. (n = 3 independent experiments); *: p < 0.05 *versus* 4.5 J/cm^2^. **Figure S2.** MB is preferentially localized in the lysosomes. (**a**) Low magnification images of the data shown in Fig. 5 of the cells simultaneously incubated with LysoTracker green (LysoTG) and MB. Merged images of the following two images (yellow; left column). MB Fluorescence (red; middle column) and fluorescence arising from LysoTracker (green; right column). (**b**) Confocal microscopy images of the cells simultaneously incubated with MitoTracker green (MitoTG) and MB. Merged images of the following two images (yellow; left column). MB Fluorescence (red; middle column) and fluorescence arising from MitoTracker (green; right column). Hoechst 33342, indicating nuclei. [MB] = 20 μM; [LysoTG] = 300 nM; [MitoTG] = 300 nM; nucleus (HO 3334 = 300 nM). Size bar: 10μm. **Figure S3.** No evidences of apoptotic nuclei after MB-PDT. (**a**) Representative nuclei of human mammary cells treated for 1 h, 3 h and 24 h with MB-PDT with 0.2 μM of MB. Nucleus stained with Hoechst 33342 (blue) and propidium iodide (red). Size bar: 20μm (**b**) Viability time curves after MB-PDT of cell cultures with 2 (a) or 20 μM of MB (b) followed by of 4.5 J/cm2 irradiation obtained after 1 h, 3 h and 24 h post-irradiation (n = 3 independent experiments) * p < 0.05 versus MCF-10A. Results are shown as mean ± s.e.m. (DOCX 2074 kb)

## Abbreviations

^1^O_2_: Singlet oxygen
2D: Bidimensional
3D: Tridimensional
AO: Acridine orange
BAX: BCL2 and BCL2-associated X protein
BCL2: B-Cell CLL/lymphoma 2
BP: Band pass
DMEM-F12: Dulbecco’s Modified Eagle’s Medium/Nutrient F-12 Ham
DTPA: Diethylene triamine penta acetic acid
ER: Estrogen receptors
GSH: Reduced glutathione
HER2: Epidermal growth factor receptor-2
HO: Hoechst 33342
HPLC: High performance liquid chromatography
LED: Light emitting diode
LP: Long pass
LSM: Laser scan microscope
LysoTG: Lysosomal marker
MB: Methylene blue
MB-PDT: PDT Using methylene blue
MitoTG: Mitochondrial marker
NIR: Near infrared
OPA: Orthophthaldehyde
PDT: Photodynamic therapy
PI: Propidium iodide
PMT: Photon multiplier tube
PR: Progesterone receptors
Ps: Photosensitizer
PVDF: Polyvinyl difluoride
RPMI: Roswell Park Memorial Institute Medium Modified
siCTR: siRNA control of scrambled sequence
TNBC: Triple-negative breast cancer

## References

[1] Siegel RL, Miller KD, Jemal A (2015). Cancer statistics, 2015. CA Cancer J Clin.

[2] Hanahan D, Weinberg R a (2011). Hallmarks of cancer: the next generation. Cell.

[3] Gomes LR, Terra LF, Sogayar MC, Labriola L (2011). Epithelial-mesenchymal transition: implications in cancer progression and metastasis. Curr Pharm Biotechnol.

[4] Cancer T, Atlas G (2012). Comprehensive molecular portraits of human breast tumours.

[5] Panzarini E, Inguscio V, Fimia GM, Dini L (2014). Rose Bengal Acetate PhotoDynamic Therapy (RBAc-PDT) induces exposure and release of Damage-Associated Molecular Patterns (DAMPs) in human HeLa cells. PLoS One.

[6] Paul A, Gunewardena S, Stecklein SR, Saha B, Parelkar N, Danley M, Rajendran G, Home P, Ray S, Jokar I (2014). PKC k / i signaling promotes triple-negative breast cancer growth and metastasis. Cell Death Differ.

[7] Eckhardt BL, Francis P a, Parker BS, Anderson RL (2012). Strategies for the discovery and development of therapies for metastatic breast cancer. Nat Rev Drug Discov.

[8] Rizvi I, Celli JP, Evans CL, Abu-Yousif AO, Muzikansky A, Pogue BW, Finkelstein D, Hasan T (2010). Synergistic enhancement of carboplatin efficacy with photodynamic therapy in a three-dimensional model for micrometastatic ovarian cancer. Cancer Res.

[9] Ahn TG, Lee BR, Choi EY, Kim DW, Han SJ. Photodynamic therapy for breast cancer in a BALB/c mouse model. J Gynecol Oncol. 2012:115-19. 10.3802/jgo.2012.23.2.115 PMC3325345 22523628

[10] Montazerabadi AR, Sazgarnia A, Bahreyni-Toosi MH, Ahmadi A, Shakeri-Zadeh A, Aledavood A (2012). Mitoxantrone as a prospective photosensitizer for photodynamic therapy of breast cancer. Photodiagnosis Photodyn Ther.

[11] Acedo P, Stockert JC, Cañete M, Villanueva A (2014). Two combined photosensitizers: a goal for more effective photodynamic therapy of cancer. Cell Death Dis.

[12] Agostinis P, Berg K, Cengel K a, Foster TH, Girotti AW, Gollnick SO, Hahn SM, Hamblin MR, Juzeniene A, Kessel D, Korbelik M, Moan J, Mroz P, Nowiz D, Piette J, Willson BC, Golab J (2011). Photodynamic therapy of cancer : an update. Am Cancer Soc.

[13] Simone CB, Friedberg JS, Glatstein E, Stevenson JP, Sterman DH, Stephen M, Cengel KA (2011). Photodynamic therapy for the treatment of non-small cell lung cancer.

[14] Maawy AA, Hiroshima Y, Zhang Y, Garcia-Guzman M, Luiken GA, Kobayashi H, Hoffman RM, Bouvet M (2014). Photoimmunotherapy lowers recurrence after pancreatic cancer surgery in orthotopic nude mouse models. J Surg Res.

[15] Maawy AA, Hiroshima Y, Zhang Y, Heim R, Makings L, Garcia-Guzman M, Luiken GA, Kobayashi H, Hoffman RM, Bouvet M (2015). Near Infra-Red Photoimmunotherapy with Anti-CEA-IR700 results in extensive tumor lysis and a significant decrease in tumor burden in orthotopic mouse models of pancreatic cancer. PLoS One.

[16] Allison RR, Sibata CH (2010). Oncologic photodynamic therapy photosensitizers: a clinical review. Photodiagnosis Photodyn Ther.

[17] Celli JP, Spring BQ, Rizvi I, Evans CL, Samkoe KS, Verma S, Pogue BW, Hasan T (2010). Imaging and Photodynamic Therapy : Mechanisms, Monitoring, and Optimization.

[18] Plaetzer K, Krammer B, Berlanda J, Berr F (2009). Photophysics and photochemistry of photodynamic therapy : fundamental aspects.

[19] Bacellar I, Tsubone T, Pavani C, Baptista M (2015). Photodynamic efficiency: from molecular photochemistry to cell death. Int J Mol Sci.

[20] Itri R, Junqueira HC, Mertins O, Baptista MS (2014). Membrane changes under oxidative stress: the impact of oxidized lipids. Biophys Rev.

[21] Tardivo JP, Adami F, Correa JA, Pinhal MAS, Baptista MS (2014). A clinical trial testing the efficacy of PDT in preventing amputation in diabetic patients. Photodiagnosis Photodyn Ther.

[22] Wagner M, Suarez ER, Theodoro TR, Machado Filho CDAS, Gama MFM, Tardivo JP, Paschoal FM, Pinhal MADS (2012). Methylene blue photodynamic therapy in malignant melanoma decreases expression of proliferating cell nuclear antigen and heparanases. Clin Exp Dermatol.

[23] Song D, Lindoso AL, Oyafuso LK, Cardoso L, Uchoa AF. Photodynamic therapy using methylene blue to treat cutaneous leishmaniasis. Photomed Laser Surg. 2011;29:711–15. 10.1089/pho.2010.2915 21671755

[24] Tardivo JP, Del Giglio A, Paschoal LH, Baptista MS (2006). New photodynamic therapy protocol to treat AIDS-related Kaposi’s sarcoma. Photomed Laser Surg.

[25] Tardivo JP, Del Giglio A, De Oliveira CS, Gabrielli DS, Junqueira HC, Tada DB, Severino D, De Fátima TR, Baptista MS (2005). Methylene blue in photodynamic therapy: From basic mechanisms to clinical applications. Photodiagnosis Photodyn Ther.

[26] Schirmer RH, Adler H, Pickhardt M, Mandelkow E (2011). “Lest we forget you — methylene blue ….” NBA.

[27] Oz M, Lorke DE, Hasan M, Petroianu GA. Cellular and molecular actions of methylene blue in the nervous system. Med Res Rev. 2011;1:93–117. 10.1002/med.20177 PMC3005530 19760660

[28] Lee GY, Kenny P a, Lee EH, Bissell MJ (2007). Three-dimensional culture models of normal and malignant breast epithelial cells. Nat Methods.

[29] Forrester HB, Vidair CA, Albright N, Ling CC, Dewey WC (1999). Using computerized video time lapse for quantifying cell death of X-irradiated rat embryo cells transfected with c-myc or c-Ha-ras. Cancer Res.

[30] Kand’ár R, Žáková P, Lotková H, Kučera O, Červinková Z (2007). Determination of reduced and oxidized glutathione in biological samples using liquid chromatography with fluorimetric detection. J Pharm Biomed Anal.

[31] Paglin S, Hollister T, Delohery T, Hackett N, McMahill M, Sphicas E, Domingo D, Yahalom J (2001). A novel response of cancer cells to radiation involves autophagy and formation of acidic vesicles. Cancer Res.

[32] Eizirik DL, Mandrup-Poulsen T (2001). A choice of death-the signal-transduction of immune-mediated beta-cell apoptosis. Diabetologia.

[33] Eizirik DL, Moore F, Flamez D, Ortis F (2008). Use of a systems biology approach to understand pancreatic β -cell death in Type 1 diabetes. Biochem Soc Trans.

[34] Nielsen K, Sparre T, Larsen MR, Nielsen M, Fey SJ, Larsen PM, Roepstorff P, Nerup J, Karlsen AE (2004). Protein expression changes in a cell system of beta-cell maturation reflect an acquired sensitivity to IL-1 β. Diabetologia.

[35] Nielsen K, Karlsen AE, Deckert M, Madsen OD, Serup P, Mandrup-poulsen T, Nerup J (1999). β-Cell maturation leads to in vitro sensitivity to cytotoxins. Diabetes.

[36] Belmokhtar C a, Hillion J, Ségal-Bendirdjian E (2001). Staurosporine induces apoptosis through both caspase-dependent and caspase-independent mechanisms. Oncogene.

[37] Narita Y, Asai A, Kuchino Y, Kirino T (2000). Actinomycin D and staurosporine, potent apoptosis inducers in vitro, are potentially effective chemotherapeutic agents against glioblastoma multiforme. Cancer Chemother Pharmacol.

[38] Galluzzi L, Bravo-San Pedro JM, Vitale I, Aaronson S a, Abrams JM, Adam D, Alnemri ES, Altucci L, Andrews D, Annicchiarico-Petruzzelli M, Baehrecke EH, Bazan NG, Bertrand MJ, Bianchi K, Blagosklonny M V, Blomgren K, Borner C, Bredesen DE, Brenner C, Campanella M, Candi E, Cecconi F, Chan FK, Chandel NS, Cheng EH, Chipuk JE, Cidlowski J a, Ciechanover a, Dawson TM, Dawson VL, et al. Essential versus accessory aspects of cell death: recommendations of the NCCD 2015. Cell Death Differ. 2014;1:1–16. 10.1038/cdd.2014.137 PMC4262782 25236395

[39] Galluzzi L, Vitale I, Abrams JM, Alnemri ES, Baehrecke EH, Blagosklonny MV, Dawson TM, Dawson VL, El-Deiry WS, Fulda S, Gottlieb E, Green DR, Hengartner MO, Kepp O, Knight R a, Kumar S, Lipton SA, Lu X, Madeo F, Malorni W, Mehlen P, Nuñez G, Peter ME, Piacentini M, Rubinsztein DC, Shi Y, Simon H-U, Vandenabeele P, White E, Yuan J (2012). Molecular definitions of cell death subroutines: recommendations of the Nomenclature Committee on Cell Death 2012. Cell Death Differ.

[40] Reiners JJ, Agostinis P, Berg K, Oleinick NL, Kessel D. Assessing autophagy in the context of photodynamic therapy. Autophagy. 2010;6:7–18. 10.4161/auto.6.1.10220 PMC2861993 19855190

[41] El-Hussein A, Mfouo-Tynga I, Abdel-Harith M, Abrahamse H (2015). Comparative study between the photodynamic ability of gold and silver nanoparticles in mediating cell death in breast and lung cancer cell lines. J Photochem Photobiol B Biol.

[42] Zhang S, Yang L, Ling X, Shao P, Wang X, Edwards WB, Bai M (2015). Tumor mitochondria-targeted photodynamic therapy with a translocator protein (TSPO)-specific photosensitizer. Acta Biomater.

[43] Stuchinskaya T, Moreno M, Cook MJ, Edwards DR, Russell D a (2011). Targeted photodynamic therapy of breast cancer cells using antibody-phthalocyanine-gold nanoparticle conjugates. Photochem Photobiol Sci.

[44] Shemesh CS, Hardy CW, Yu DS, Fernandez B, Zhang H (2014). Indocyanine green loaded liposome nanocarriers for photodynamic therapy using human triple negative breast cancer cells. Photodiagnosis Photodyn Ther.

[45] Oliveira CS, Turchiello R, Kowaltowski AJ, Indig GL, Baptista MS (2011). Free Radical Biology & Medicine Major determinants of photoinduced cell death : Subcellular localization versus photosensitization ef fi ciency. Free Radic Biol Med.

[46] Traverso N, Ricciarelli R, Nitti M, Marengo B, Furfaro AL, Pronzato MA, Marinari UM, Domenicotti C (2013). Role of glutathione in cancer progression and chemoresistance. Oxid Med Cell Longev.

[47] Pelicano H, Zhang W, Liu J, Hammoudi N, Dai J, Xu R-H, Pusztai L, Huang P (2014). Mitochondrial dysfunction in some triple-negative breast cancer cell lines: role of mTOR pathway and therapeutic potential. Breast Cancer Res.

[48] Guan J, Lai X, Wang X, Leung AW, Zhang H, Xu C (2014). Photodynamic action of methylene blue in osteosarcoma cells in vitro. Photodiagnosis Photodyn Ther.

[49] Lim EJ, Oak CH, Heo J, Kim YH (2013). Methylene blue-mediated photodynamic therapy enhances apoptosis in lung cancer cells. Oncol Rep.

[50] Xue LY, Chiu SM, Oleinick NL (2001). Photodynamic therapy-induced death of MCF-7 human breast cancer cells: a role for caspase-3 in the late steps of apoptosis but not for the critical lethal event. Exp Cell Res.

[51] Hoi SW-H, Wong HM, Chan JY-W, Yue GGL, Tse GM-K, Law BK-B, Fong WP, Fung KP (2012). Photodynamic therapy of Pheophorbide a inhibits the proliferation of human breast tumour via both caspase-dependent and -independent apoptotic pathways in in vitro and in vivo models. Phytother Res.

[52] Devarajan E, Sahin AA, Chen JS, Krishnamurthy RR, Aggarwal N, Brun A-M, Sapino A, Zhang F, Sharma D, Yang X-H, Tora AD, Mehta K (2002). Down-regulation of caspase 3 in breast cancer: a possible mechanism for chemoresistance. Oncogene.

[53] Galluzzi L, Bravo-San Pedro JM, Kroemer G (2014). Organelle-specific initiation of cell death. Nat Cell Biol.

[54] Xu DD, Lam HM, Hoeven R, Xu CB, Leung AW, Cho WCS (2013). Photodynamic therapy induced cell death of hormone insensitive prostate cancer PC-3 cells with autophagic characteristics.

[55] Panzarini E, Inguscio V, Dini L (2011). Timing the multiple cell death pathways initiated by Rose Bengal acetate photodynamic therapy. Cell Death Dis.

[56] Xue L, Chiu S, Azizuddin K, Joseph S, Oleinick NL (2007). The Death of Human Cancer Cells Following Photodynamic Therapy : Apoptosis Competence is Necessary for Bcl-2 Protection but not for Induction of Autophagy †.

[57] Jain K, Paranandi KS, Sridharan S, Basu A (2013). Autophagy in breast cancer and its implications for therapy. Am J Cancer Res.

[58] Kreuzaler P, Watson CJ (2012). Killing a cancer : what are the alternatives ?. Nat Rev Cancer.

[59] Nandakumar V, Kelbauskas L, Hernandez KF, Lintecum KM, Senechal P, Bussey KJ, Davies PCW, Johnson RH, Meldrum DR. Isotropic 3D nuclear morphometry of normal, fibrocystic and malignant breast epithelial cells reveals new structural alterations. PLoS One. 2012;7:1-9. 10.1371/journal.pone.0029230 PMC3252316 22242161

[60] Lo AT, Mori H, Mott J, Bissell MJ (2012). Constructing three-dimensional models to study mammary gland branching morphogenesis and functional differentiation. J Mammary Gland Biol Neoplasia.

[61] Kenny PA, Lee GY, Myers CA, Neve RM, Semeiks JR, Spellman PT, Lorenz K, Lee EH, Barcellos-Hoff MH, Petersen OW, Gray JW, Bissell MJ (2007). The morphologies of breast cancer cell lines in three-dimensional assays correlate with their profiles of gene expression. Mol Oncol.

[62] Bissell MJ, Ghajar CM, Lee LP (2012). From single cells to biology. Integr Biol.

