# Supplementary material for: Methylene blue photodynamic therapy induces selective and massive cell death in human breast cancer cells
Source: BMC Cancer. 2017 Mar 15;17:194. doi: 10.1186/s12885-017-3179-7 (PMC5353937; doi:10.1186/s12885-017-3179-7)
Supplement: Additional file 1: — Figure S1. MB-PDT induced massive cell death in MDA-MB-231 without dark cytotoxicity. (a) Representative graph showing the viability of MCF-10A, MCF-7 and MDA-MB-231 cells after MB-PDT with 20 μM of MB followed by 4.5 J/cm2 irradiation and the experimental controls: irradiation alone (λ); dark toxicity of MB, that means incubation without irradiation (MB); or not treated cells (control). These results were obtained after 24 h (n = 3 independent experiments); *: p < 0.05 versus control. (b) Viability of MDA-MB-231 cells after MB-PDT with MB (20 μM) followed by 4.5 J/cm2 or 1.5 J/cm2 irradiation after 24 h. Results are shown as mean ± s.e.m. (n = 3 independent experiments); *: p < 0.05 versus 4.5 J/cm2. Figure S2. MB is preferentially localized in the lysosomes. (a) Low magnification images of the data shown in Fig. 5 of the cells simultaneously incubated with LysoTracker green (LysoTG) and MB. Merged images of the following two images (yellow; left column). MB Fluorescence (red; middle column) and fluorescence arising from LysoTracker (green; right column). (b) Confocal microscopy images of the cells simultaneously incubated with MitoTracker green (MitoTG) and MB. Merged images of the following two images (yellow; left column). MB Fluorescence (red; middle column) and fluorescence arising from MitoTracker (green; right column). Hoechst 33342, indicating nuclei. [MB] = 20 μM; [LysoTG] = 300 nM; [MitoTG] = 300 nM; nucleus (HO 3334 = 300 nM). Size bar: 10μm. Figure S3. No evidences of apoptotic nuclei after MB-PDT. (a) Representative nuclei of human mammary cells treated for 1 h, 3 h and 24 h with MB-PDT with 0.2 μM of MB. Nucleus stained with Hoechst 33342 (blue) and propidium iodide (red). Size bar: 20μm (b) Viability time curves after MB-PDT of cell cultures with 2 (a) or 20 μM of MB (b) followed by of 4.5 J/cm2 irradiation obtained after 1 h, 3 h and 24 h post-irradiation (n = 3 independent experiments) * p < 0.05 versus MCF-10A. Results are shown as mean ± s.e.m. ( [file 12885_2017_3179_MOESM1_ESM.docx]

**Additional file: Figure S1:**


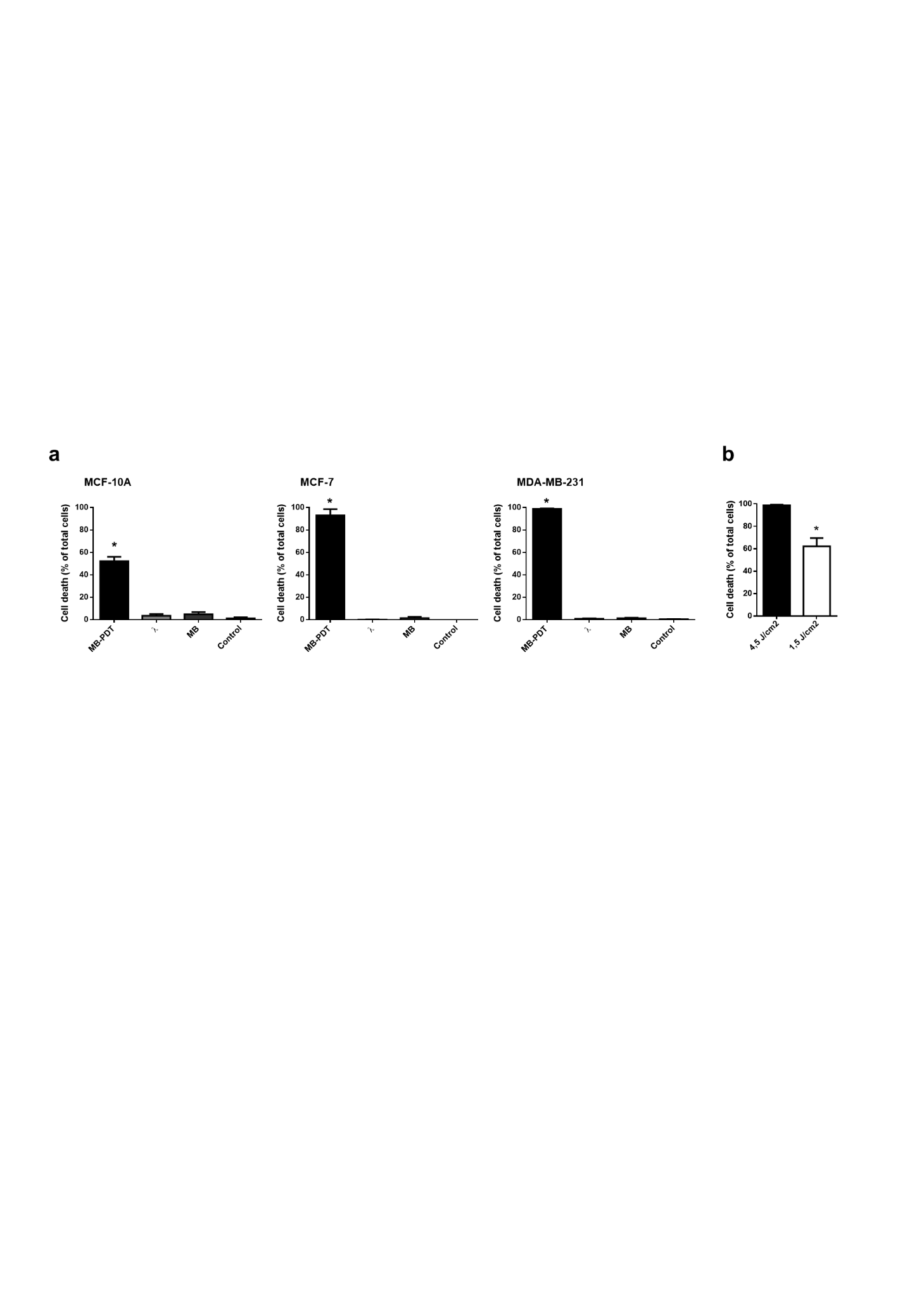


**Additional file 1: Figure S1. MB-PDT induced massive cell death in MDA-MB-231 without dark cytotoxicity.** (a) Representative graph showing the viability of MCF-10A, MCF-7 and MDA-MB-231 cells after MB-PDT with 20 µM of MB followed by 4.5 J/cm^2^ irradiation and the experimental controls: irradiation alone (λ); dark toxicity of MB, that means incubation without irradiation (MB); or not treated cells (control). These results were obtained after 24 hours (n=3 independent experiments); *: p<0.05 *versus* control. (b) Viability of MDA-MB-231 cells after MB-PDT with MB (20 µM) followed by 4.5 J/cm^2^ or 1.5 J/cm^2^ irradiation after 24 h. Results are shown as mean ±s.e.m. (n=3 independent experiments); *: p<0.05 *versus* 4.5 J/cm^2^

**Additional file 2: Figure S2:**


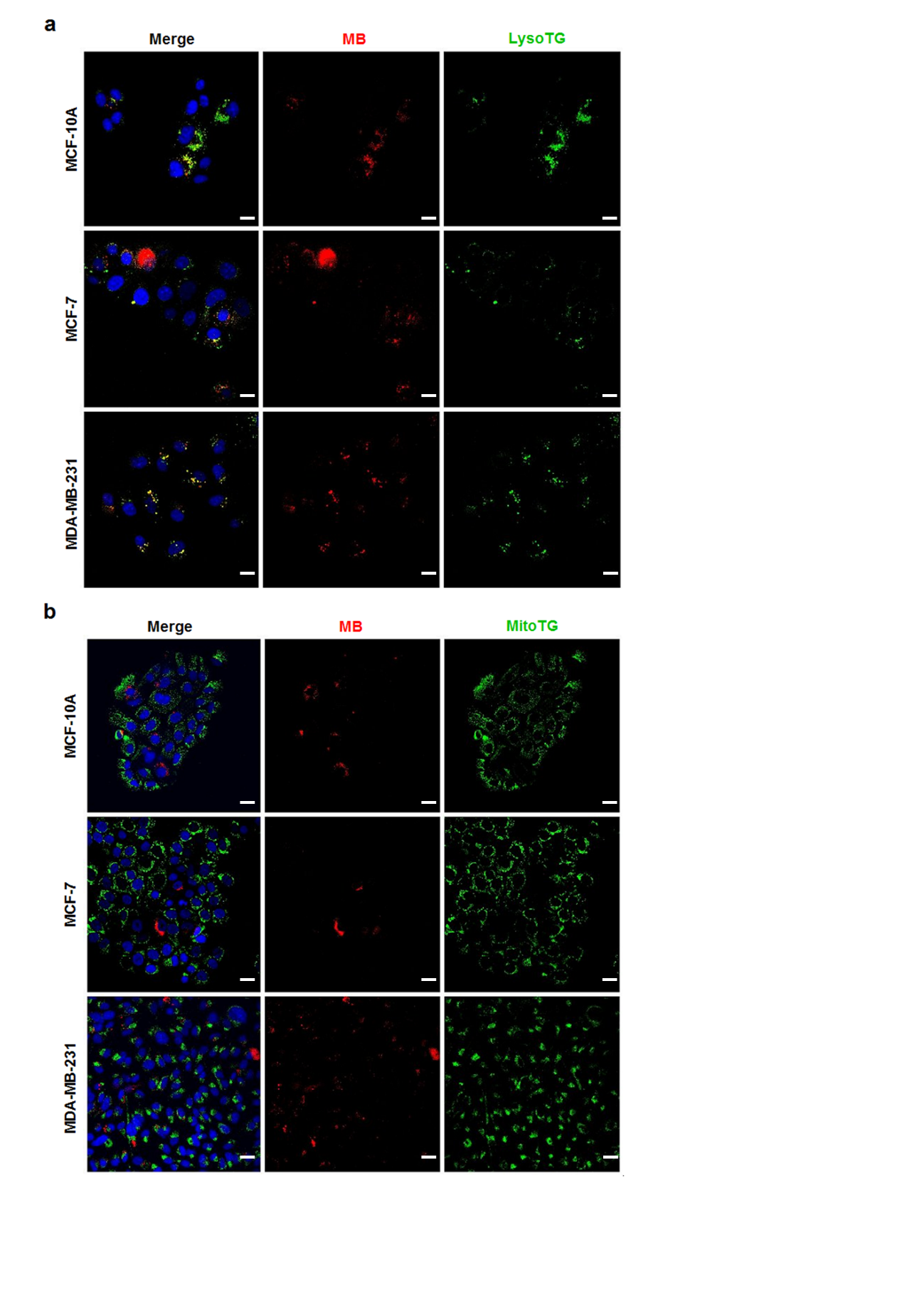


**Additional file 2: Figure S2. MB is preferentially localized in the lysosomes.** (a) Low magnification images of the data shown in figure 5 of the cells simultaneously incubated with LysoTracker green (LysoTG) and MB. Merged images of the following two images (yellow; left column). MB Fluorescence (red; middle column) and fluorescence arising from LysoTracker (green; right column). (b) Confocal microscopy images of the cells simultaneously incubated with MitoTracker green (MitoTG) and MB. Merged images of the following two images (yellow; left column). MB Fluorescence (red; middle column) and fluorescence arising from MitoTracker (green; right column). Hoechst 33342, indicating nuclei. [MB] = 20 µM; [LysoTG] = 300 nM; [MitoTG] = 300 nM; nucleus (HO 3334 = 300 nM). Size bar: 10µm.

**Additional file 3: Figure S3:**
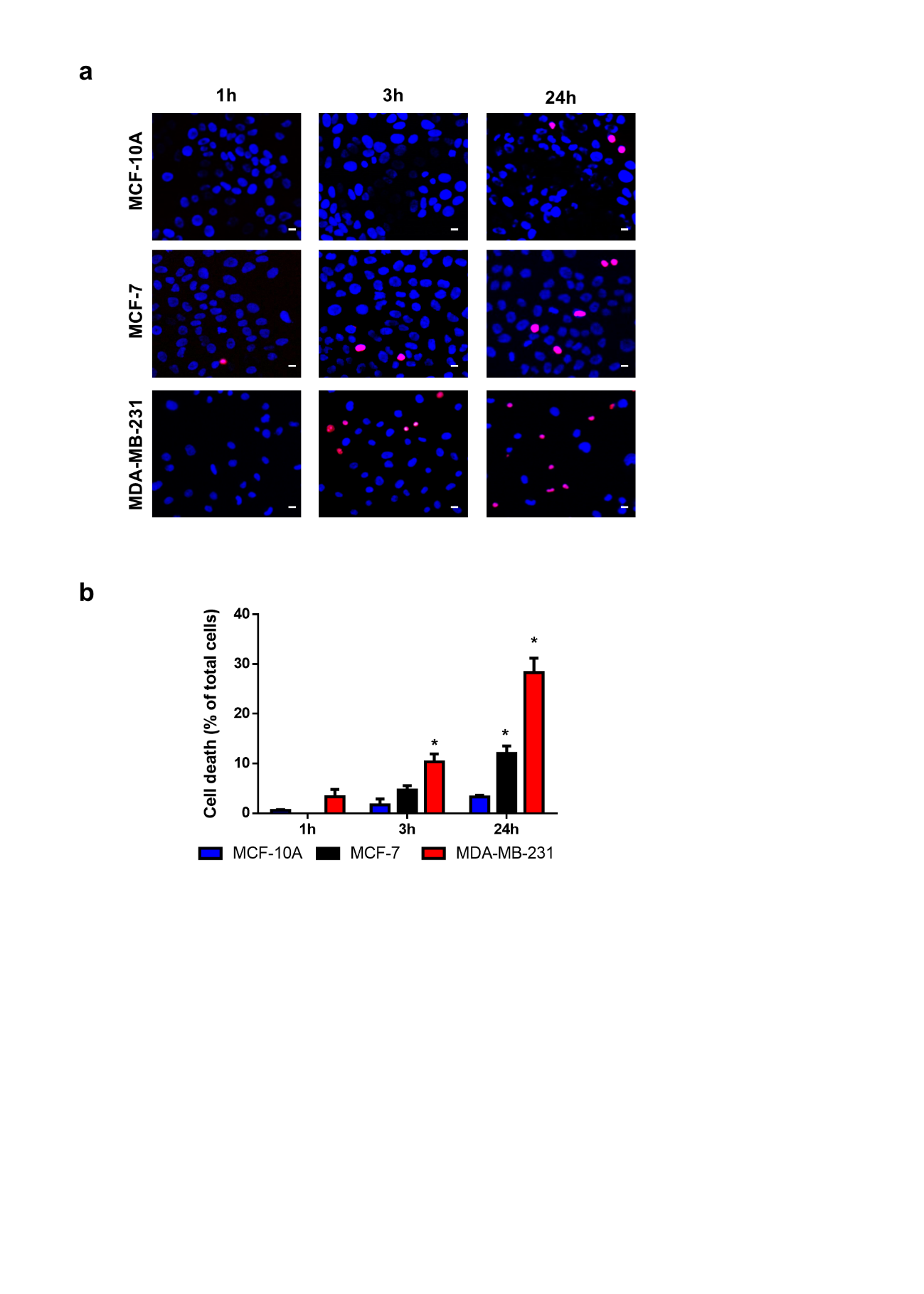


**Additional file 3: Figure S3. No evidences of apoptotic nuclei after MB-PDT.** (a) Representative nuclei of human mammary cells treated for 1 h, 3 h and 24 h with MB-PDT with 0.2 µM of MB. Nucleus stained with Hoechst 33342 (blue) and propidium iodide (red). Size bar: 20µm (b) Viability time curves after MB-PDT of cell cultures with 2 (a) or 20 µM of MB (b) followed by of 4.5 J/cm2 irradiation obtained after 1 h, 3 h and 24 h post-irradiation (n=3 independent experiments) * p<0.05 versus MCF-10A. Results are shown as mean ±s.e.m.
